# TRPM8-Dependent Dynamic Response in a Mathematical Model of Cold Thermoreceptor

**DOI:** 10.1371/journal.pone.0139314

**Published:** 2015-10-01

**Authors:** Erick Olivares, Simón Salgado, Jean Paul Maidana, Gaspar Herrera, Matías Campos, Rodolfo Madrid, Patricio Orio

**Affiliations:** 1 Centro Interdisciplinario de Neurociencia de Valparaíso, Universidad de Valparaíso, Valparaíso, Chile; 2 Facultad de Química y Biología, Universidad de Santiago de Chile, Santiago, Chile; 3 Instituto de Neurociencia, Facultad de Ciencias, Universidad de Valparaíso, Valparaíso, Chile; University of South California, UNITED STATES

## Abstract

Cold-sensitive nerve terminals (CSNTs) encode steady temperatures with regular, rhythmic temperature-dependent firing patterns that range from irregular tonic firing to regular bursting (static response). During abrupt temperature changes, CSNTs show a dynamic response, transiently increasing their firing frequency as temperature decreases and silencing when the temperature increases (dynamic response). To date, mathematical models that simulate the static response are based on two depolarizing/repolarizing pairs of membrane ionic conductance (slow and fast kinetics). However, these models fail to reproduce the dynamic response of CSNTs to rapid changes in temperature and notoriously they lack a specific cold-activated conductance such as the TRPM8 channel. We developed a model that includes TRPM8 as a temperature-dependent conductance with a calcium-dependent desensitization. We show by computer simulations that it appropriately reproduces the dynamic response of CSNTs from mouse cornea, while preserving their static response behavior. In this model, the TRPM8 conductance is essential to display a dynamic response. In agreement with experimental results, TRPM8 is also needed for the ongoing activity in the absence of stimulus (i.e. neutral skin temperature). Free parameters of the model were adjusted by an evolutionary optimization algorithm, allowing us to find different solutions. We present a family of possible parameters that reproduce the behavior of CSNTs under different temperature protocols. The detection of temperature gradients is associated to a homeostatic mechanism supported by the calcium-dependent desensitization.

## Introduction

In mammals, cold is detected by specific cutaneous thermoreceptor neurons of the somatosensory system. The transduction of cold stimuli into electrical impulses takes place in the free endings of the thermoreceptor fibers, corresponding to the axonal endings of cold-sensitive neurons from trigeminal and dorsal root ganglion [1,2].

Cold thermoreceptors frequently have spontaneous firing of action potentials at resting skin temperature (33–34°C). Their response to temperature displays two essential features: The dynamic response, evoked by a change in temperature; and the static response, which is the firing pattern reached after the terminal adapts to a new stationary temperature [3]. A decrease in temperature causes a marked increase in the firing frequency, often in the form of burst firing. Conversely, if the temperature increases, the firing frequency drops to zero, and the nerve ending starts firing again when a new static firing pattern is reached [4,5].

The static response of cold thermoreceptors consists of several firing patterns that depend on the temperature at which the terminal is held. At resting skin temperature, they show a regular beating discharge of electrical impulses. At lower temperatures bursting is promoted, making single spike events less likely, and the interval between events is increased. On the contrary, at temperatures above ~34°C there is evidence of skipping of events provided by intervals that are twice (or higher multiples) the mean [3].

In an attempt to understand the origin of the static firing patterns, several mathematical models that reproduce them have been proposed [6–8]. Based on a general model of slow wave or parabolic bursting [9], regular spiking or bursting is driven by slow membrane oscillations, on top of which fast voltage-activated sodium and potassium channels generate action potentials. When the usual effect of temperature on ion channels is considered (Q_10_~1.3 for ion currents and Q_10_~3 for ion channel kinetics [10]), the models show the different firing patterns as temperature changes. Low temperature lengthens the period of the oscillation, allowing more action potentials per cycle. The proposed molecular counterparts involved in the generation of the slow oscillation include slow TTX-resistant persistent sodium currents [11,12] and low-threshold calcium channels [13,14]. The HCN1 channels are also related to the phenomenon, helping to set the correct frequency of the underlying slow oscillation [15,16].

However, the mathematical models mentioned above only reproduce the firing patterns observed when the terminal is already adapted to a steady temperature. They do not show an important change in the firing frequency upon cooling or heating, neither they discriminate between temperature increase and decrease. In other words, they only reproduce the static response and lack the dynamic response. Coincidentally, the current models do not take into account the contribution of the TRPM8 ion channel.

The TRPM8 channel is considered the main molecular entity responsible for the detection of cold temperatures in the somatosensory system [1,17,18]. This channel behaves as a polymodal receptor, being activated by membrane depolarization, cold, and chemicals that evoke cooling sensations such as menthol and icilin [19]. Activation of TRPM8 channels induce increases in firing probability due to an inward current that depolarizes the cell [20–22]. Knock-out mice lacking functional expression of the channel present a strongly reduced cold sensitivity [23–25]. Cold- or menthol-induced TRPM8 current undergoes desensitization, a phenomenon that is dependent on extracellular calcium and that involves activation of phospholipase-C and depletion of phosphatidylinositol 4,5-bisphosphate (PIP_2_) in the membrane [21,26–28]. Besides, a Ca^2+^-calmodulin dependent mechanism also contributes to the desensitization of TRPM8 current [29]. The time scale of the current desensitization is the same as the adaptation of the response to temperature changes of cold-sensitive neurons, suggesting that as TRPM8 current declines, the terminal decreases its firing rate and adapts to a new temperature.

Here, we test the hypothesis that the cold-induced activation of TRPM8 and its following activity-dependent desensitization can underlie most of the features of the dynamic response observed in cold-sensitive nerve endings. In order to do that, we introduced equations describing TRPM8 activity into an existing model of cold receptor that reproduces the static response [6]. We found that the contribution of TRPM8 to the dynamic response is far from simple, as the behavior of cold-sensitive neurons involves the participation of several ion channels. Nevertheless, with the appropriate set of parameters our model reproduces the characteristic responses to changes in temperature while at the same time maintaining the different firing patterns observed in the static response. We also show that variations in some parameters can originate different ‘phenotypes’ of models, resembling the variability observed in experimental recordings. By manipulating the degree of TRPM8 desensitization, we found that during the static response the model lies in a region with a moderate to low firing rate, thus allowing the transit either to a high firing rate (during cooling) or to the absence of firing (during heating). Our model also reproduces experimental results showing that the spontaneous activity of cold thermoreceptors is dependent on the presence of the TRPM8 current [30]. We provide the first model of cold thermoreceptors that reproduces both the dynamic and static electrical responses to temperature.

## Methods

### Animals

Studies were performed on young adult BalbC mice (P30-P40). All experiments were conducted according to bioethical guidelines of the Comisión Nacional de Investigación Científica y Tecnológica de Chile (CONICYT) and have been approved by the Bioethical Committee of the Universidad de Santiago de Chile. Animals were killed by exposure to CO_2_ and decapitated. Eyes were carefully removed and placed in the recording chamber.

### Extracellular recording of single nerve terminal impulses

Firing of cold thermoreceptor neurons from mouse cornea was recorded as in [30]. Briefly, the excised eyes were placed in a small recording chamber and secured by application of slight suction to a tube connected to the conical bottom. The isolated eyes were continuously perfused (1 ml/min) with a solution containing (in mM): 128NaCl, 5 KCl, 1 NaH_2_PO_4_, 26 NaHCO_3_, 2.4 CaCl_2_, 1.3 MgCl_2_ and 10 glucose. The solution was gassed with a mix of O_2_ (95%) and CO_2_ (5%) and maintained at the desired temperature with a computer-controlled Peltier device, located at the entrance of the chamber. To record nerve terminal activity, glass micropipette electrodes (tip diameter ~50 μm) filled with the same solution were applied to the corneal surface with light suction. Cold thermoreceptors were identified by their spontaneous discharge at 33°C, which is increased by cooling of the superfusing solution. Voltage signals were amplified with an AC amplifier (A-M Systems 1800; gain 10,000; low cut-off 1Hz; high cut-off 10 KHz). Data were captured at 33.3 KHz with a Digidata 1322a interface, coupled to a computer running pClamp 9 software (Molecular Devices). Temperature was measured at the corneal surface using a BAT-12 microprobe thermometer (Physitemp) supplemented with a IT-18 thermocouple, and acquired simultaneously with voltage signals.

### Mathematical model

The basis of our model is the Huber & Braun’s model of cold receptor [6] that reproduces the static response. To this model, we added the TRPM8 current. The equation for the membrane voltage *V* is

$$C_{m}\frac{dV}{dt}=-I_{sd}-I_{sr}-I_{d}-I_{r}-I_{M8}-I_{l}+I_{wn}$$ (1)

Where *C*_*m*_ is the membrane capacitance; *I*_*d*_, *I*_*r*_, *I*_*sd*_ and *I*_*sr*_ are the depolarizing, repolarizing, slow depolarizing and slow repolarizing currents, respectively; *I*_*M*8_ is the current mediated by the TRPM8 channel, *I*_*leak*_ corresponds to a unspecific leak current and *I*_*wn*_ is a noise term. Currents are described as:

$$I_{i}=\rho \left(T\right)g_{i}a_{i}\left(V-E_{i}\right)\;i=sd,d,r$$ (2)

$$I_{sr}=\rho \left(T\right)g_{sr}\left(\frac{a_{sr}^{2}}{a_{sr}^{2}+0.4^{2}}\right)\left(V-E_{sr}\right)$$ (3)

$$I_{M8}=g_{M8}a_{M8}\left(V-E_{M8}\right)$$ (4)

$$I_{l}=g_{l}\left(V-E_{l}\right)$$ (5)

Where *a* is an activation term that represents the open probability of the channels, *g* is the maximal conductance density, *E* is the reversal potential and *p*(*T*) is a temperature dependent correction factor for the current. (Eq 3) uses a saturating function of *a*_*sr*_ as in [16] and in agreement with the calcium dependence of small conductance calcium-dependent channels SK [31].

The activation terms *a*_*r*_ and *a*_*sd*_ follow the differential equations

$$\frac{da_{i}}{dt}=\phi \left(\text{T}\right)\frac{a_{i}^{\infty }\left(V\right)-a_{i}}{\tau_{i}};\;a_{i}^{\infty }\left(V\right)=\frac{1}{1+\text{exp}\left(-s_{i}\left(V-V_{i}^{h}\right)\right)};\;i=r,sd$$ (6)

Where *τ* is the time constant for current relaxation, *ϕ*(*T*) is a temperature factor of channel kinetics, and *a* ^∞^ is a sigmoid function of voltage with parameters *s* and *V* ^*h*^.

*a*_*sr*_ increases with the inward (negative) current *I*_*sd*_ and decays with a constant *κ*.

$$\frac{da_{sr}}{dt}=\frac{\phi \left(\text{T}\right)(-\eta I_{sd}-\kappa a_{sr})}{\tau_{sr}}$$ (7)

The parameter *η* relates the slow depolarizing current with the increase in *a*_*sr*_.

The activation of the fast depolarizing (*d*) and TRPM8 currents are faster than the typical membrane time constant and therefore are considered to be instantaneous. Their activation terms *a* are given by

$$a_{d}=a_{d}^{\infty }\left(V\right)=\frac{1}{1+\text{exp}\left(-s_{d}\left(V-V_{d}^{h}\right)\right)}$$ (8)

$$a_{M8}=a_{M8}^{\infty }\left(V,T\right)=\frac{1}{1+\text{exp}\left(-\frac{z_{M8}F}{RT}\left(V-V_{h}\left(T\right)-\delta V\right)\right)}$$ (9)

$V_{d}^{h}$ and *s*_*d*_ are the activation parameters of the *d* current. For the TRPM8 channel we use the simple two-state model proposed by Voets and colleagues [32,33] where *z*_*M*8_ is the voltage dependency. *F*, *R*, *T* are Faraday’s constant, gas constant and temperature, respectively. The voltage for half-activation *V*_*h*_ is a function of temperature:

$$V_{h}\left(T\right)=\frac{CRT-\Delta \text{E}}{z_{M8}F}$$ (10)

Where *C* is a constant related to the pre-exponential factors of the rate constants, and Δ*E* is the difference between the activation energies for channel closing and opening. We introduced an additional shift δ*V* that depends on intracellular calcium according to the equations

$$\frac{d\delta V}{dt}=\frac{\delta V^{\infty }\left(Ca^{2+}\right)-\delta V}{\tau_{\delta V}}$$ (11)

$$\delta V^{\infty }\left(Ca^{2+}\right)=\delta V_{min}+\frac{\left(\delta V_{max}-\delta V_{min}\right)\left[Ca^{2+}\right]}{\left[Ca^{2+}\right]+K_{Ca,M8}}$$ (12)

The intracellular calcium concentration is modeled considering that calcium influx to the cell is a fraction of *I*_*M*8_ and calcium removal follows a simple decay:

$$\frac{d\left[Ca^{2+}\right]}{dt}=-\frac{p_{Ca}I_{M8}}{2Fd}-\frac{\left[Ca^{2+}\right]}{\tau_{Ca}}$$ (13)

*d* is the diameter or depth of the calcium diffusion shell and *τ*_*Ca*_ is the time constant for calcium removal.

The temperature-dependent factors for conductance and kinetics are given, respectively, by:

$$\begin{array}{l} \rho \left(T\right)=1.3^{\frac{T-25}{10}}\\ \phi \left(T\right)=3.0^{\frac{T-25}{10}} \end{array}$$ (14)

Finally, the noise term was implemented as a low-pass filtered white noise using an Ornstein-Uhlenbeck process with mean 0:

$$\frac{dI_{wn}}{dt}=\frac{-I_{wn}+D\xi \left(t\right)}{\tau_{wn}}$$ (15)

Where *ξ*(*t*) is a normally distributed random variable (white noise) with zero mean and variance = 1.

The following parameters were fixed: *E*_*sd*_ = *E*_*d*_ = 50 *mV*; *E*_*sr*_ = *E*_*r*_ = −90 *mV*; *E*_*M*8_ = 0 *mV*; *E*_*l*_ = −70 *mV*;

*τ*_*sd*_ = 10 *ms*; *τ*_*sr*_ = 24 *ms*; *τ*_*r*_ = 1.5 *ms*; *s*_*sd*_ = 0.1 *mV* ^−1^; *s*_*d*_ = *s*_*r*_ = 0.25 *mV* ^−1^; $V_{sd}^{h}$ = −40 *mV*; $V_{d}^{h}=V_{r}^{h}$ = −25 *mV; η* = 0.012 *cm* ^2^/*μA*; *κ* = 0.17

*z*_*M*8_ = 0.65; *C* = 67; Δ*E* = 9000 *joule*; *K*_*Ca*,*M*8_ = 500 *nM*; d = 1 *μm; D* = 0.5 *μA*/*cm* ^2^; *τ*_*wn*_ = 1 *ms*.

*z*_*M*8_ was set to 0.65 after Table 2 in [34]. The pre-exponential factors *A* and *B* found in Eq 1 of [32] were replaced by *C* = log(*B*/*A*) ≈ 67.

The remaining parameters *g*_*M8*_, *g*_*sd*_, *g*_*sr*_, *g*_*d*_, *g*_*r*_, *g*_*l*_, *τ*_*Ca*_, *τ*_*δV*_, *p*_*Ca*_, *δV*_*min*_, *δV*_*max*_ were set as free parameters and fitted as described below until a desired behavior was found.

### Parameter fitting

To search for proper values of the free parameters, we employed a multi-objective evolutionary strategy [35]. Using an idealized cold pulse as stimulus, the objectives to be minimized consisted in: i) a basal firing rate at 33.5°C between 3.5 and 8.5 spikes/second, ii) a maximum firing rate during the cold pulse between 25 and 45 spikes/second, and iii) a firing rate equal to 0 during at least 15 seconds following the temperature descent. For each condition, the error function was 0 when it was satisfied; otherwise it increased as it leaved the boundaries.

With the described strategy, multiple solutions were found that satisfied the criteria. An initial list of 360 parameter combinations was reduced to 20 by clustering them in the parameter space with an Affinity Propagation algorithm. Only one parameter combination was selected as representative of each cluster. Table 1 lists the values of the 20 parameter combinations presented in this work.

**Table 1:** Values of free parameters that reproduce satisfactorily both the dynamic and static responses of cold sensitive nerve endings.

Paramset #	*g*_*M*8_	*g*_*sd*_	*g*_*sr*_	*g*_*d*_	*g*_*r*_	*g*_*l*_	*g*_*Ca*_	*τ*_*δV*_	*p*_*Ca*_	*δV*_*min*_	*δV*_*max*_
	[mS/cm^2^]						[ms]		×10^−4^	[mV]	
7	3.0	0.29	0.2	3.7	5.0	0.27	23400	1300	1.8	-160	215
28	2.0	0.28	0.22	3.5	4.9	0.24	27500	1250	2.5	-220	170
54	0.7	0.35	0.31	3	4.4	0.21	24000	3100	1.3	-230	250
92	0.5	0.21	0.28	4.0	4.9	0.17	14000	8200	4.7	-250	110
103	0.7	0.20	0.28	3.9	4.7	0.16	14000	9600	5.2	-225	150
134	2.5	0.30	0.25	4.0	5.0	0.24	20000	1300	3.5	-230	185
157	4.9	0.25	0.21	3.9	5.0	0.22	40000	3500	3.2	-150	170
158	1.0	0.28	0.26	3.8	4.7	0.21	26000	4000	3.6	-250	150
168	4.6	0.32	0.20	2.8	4.9	0.27	23500	5000	3.4	-190	235
185	4.4	0.33	0.21	3.0	4.7	0.26	39000	9200	3.3	-220	250
212	4.2	0.21	0.23	2.5	3.4	0.18	24500	7000	4.6	-230	240
215	2.2	0.21	0.22	2.7	3.0	0.19	19000	15000	4.7	-230	250
227	2.0	0.21	0.20	2.4	2.3	0.20	24000	8300	5.5	-250	230
272	2.0	0.33	0.21	2.7	4.6	0.27	24000	5100	1.9	-130	240
275	2.0	0.34	0.20	3.3	4.7	0.28	38000	4100	1.4	-140	240
289	1.5	0.34	0.20	3.0	4.2	0.29	21500	1400	4.8	-210	170
293	2.2	0.34	0.20	3.1	5.0	0.28	18000	5400	3.8	-150	190
311	2.6	0.33	0.21	2.8	3.7	0.27	16000	9100	5.4	-140	170
323	2.4	0.25	0.20	4.0	5.0	0.23	19000	6250	5.8	-220	170
339	4.7	0.25	0.20	4.0	5.0	0.23	19000	6200	5.8	-250	250

### Numerical simulation and analysis

The model was implemented in the Neuron simulation environment [36] and run from Python scripts [37]. Data analysis and plotting was performed with Python and the libraries Numpy, Scipy, Matplotlib and Scikit-learn.

## Results

**Fig 1:**
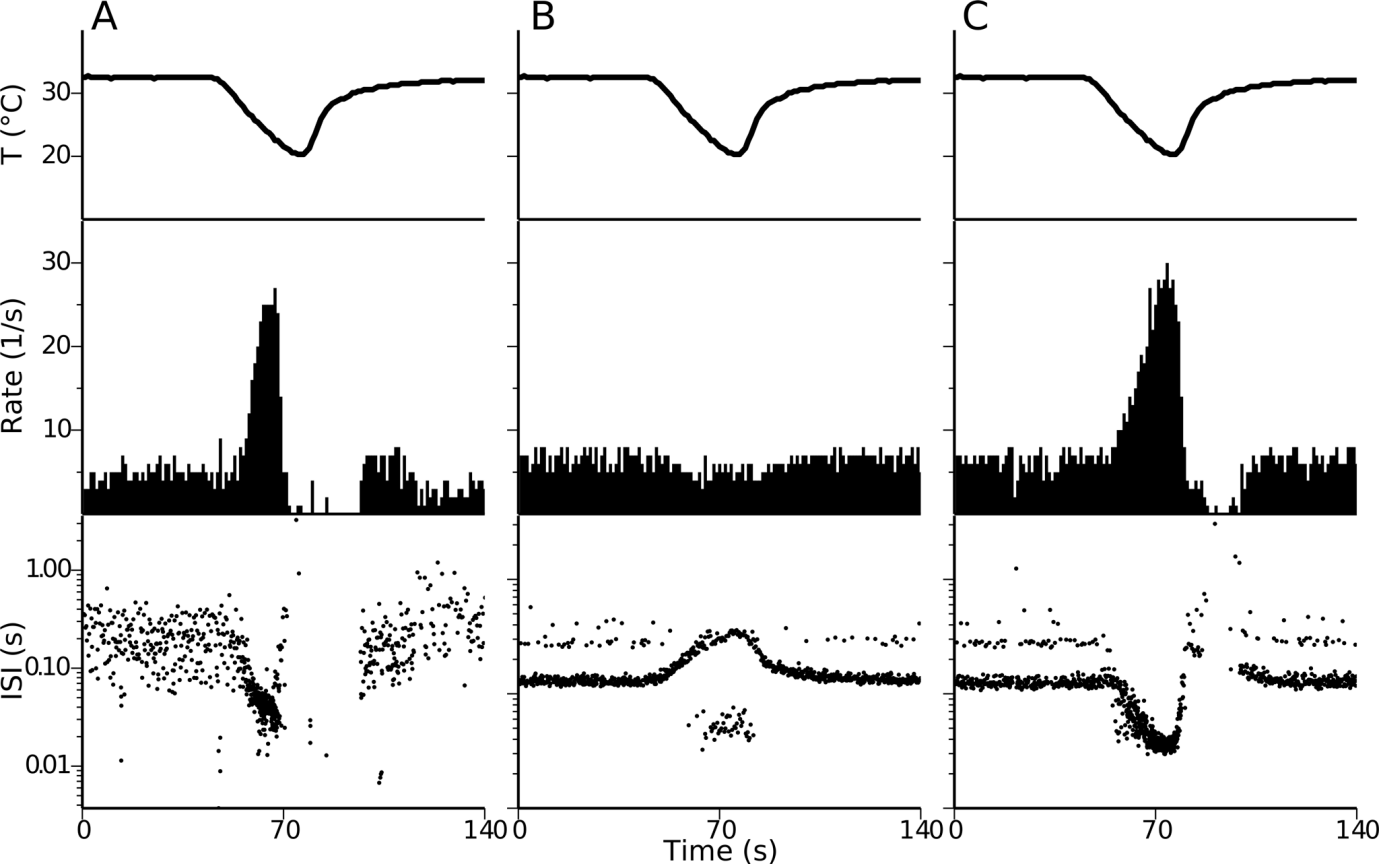
Dynamic response to cold in CS nerve endings and models. Experimental recording (A), simulation of the Huber & Braun model (B), and simulation of our model (C) exposed to the same temperature protocol (top). Middle panels show the firing rate (spikes/sec) and the bottom panels show the inter-spike intervals (log scale). Our model reproduces three main features of the experimental recording: The steady-state firing rate at the resting temperature of the skin; the increase in the firing rate in response to a temperature drop; and a silent period when the temperature is rising back. Parameter set is 92 (Table 1).

**Fig 2:**
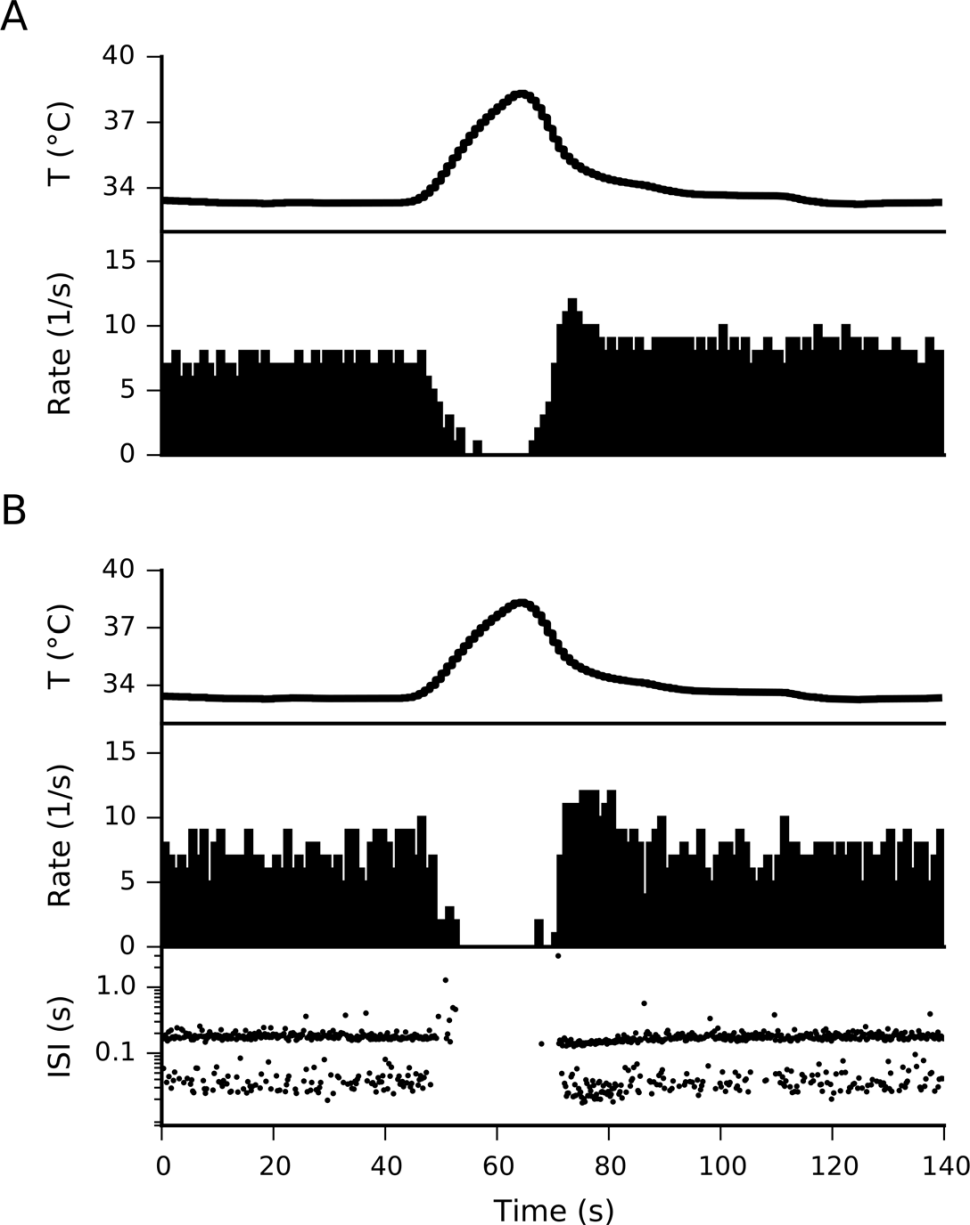
Dynamic response to heat. A. Temperature trace (top) and firing rate (bottom) in an experimental recording in mouse cornea. B. Our model subjected to the same temperature trace, showing the firing rate and ISIs obtained with parameter set 293.

Fig 1A shows the response, in firing rate and inter-spike intervals (ISIs), of a cold-sensitive nerve ending from mouse cornea subjected to the depicted temperature drop. This is a representative recording from 11 that we selected for comparison purposes in this study, although the response of corneal nerve endings to cold has already been described in mice [16,30]. In this particular set, nerve endings show a basal firing rate of 4.4 ± 1.8 spikes/sec (mean ± SD) which increase upon cooling to 35.8 ± 15.1 spikes/sec. Afterwards, while the temperature goes back to basal, the nerve endings stay silent (no spikes) for 44.2 ± 13.7 seconds. When the same stimulus is applied in a simulation of the original model of cold thermoreceptors [6], the firing rate shows no increase although the firing pattern changes (Fig 1B). On the other hand, our proposed model, reproduces satisfactorily several landmarks of the dynamic response seen in cold thermoreceptors. When the temperature stimulus is simulated into our model with TRPM8 (Fig 1C), there is an important increase in firing rate as the temperature decreases, firing as much as 30 spikes/second. When the temperature is increased back to the resting value, the spiking stops for several seconds before returning to the basal activity typical for 33°C. Cold thermoreceptors also display a characteristic silencing when the temperature raises from the basal level [38,5], a feature also reproduced by the model (Fig 2). Thus, our model can detect both temperature decreases (with a transient increase in firing rate) and temperature increases (with a decrease in firing rate), just as experimentally recorded nerve endings do.

**Fig 3:**
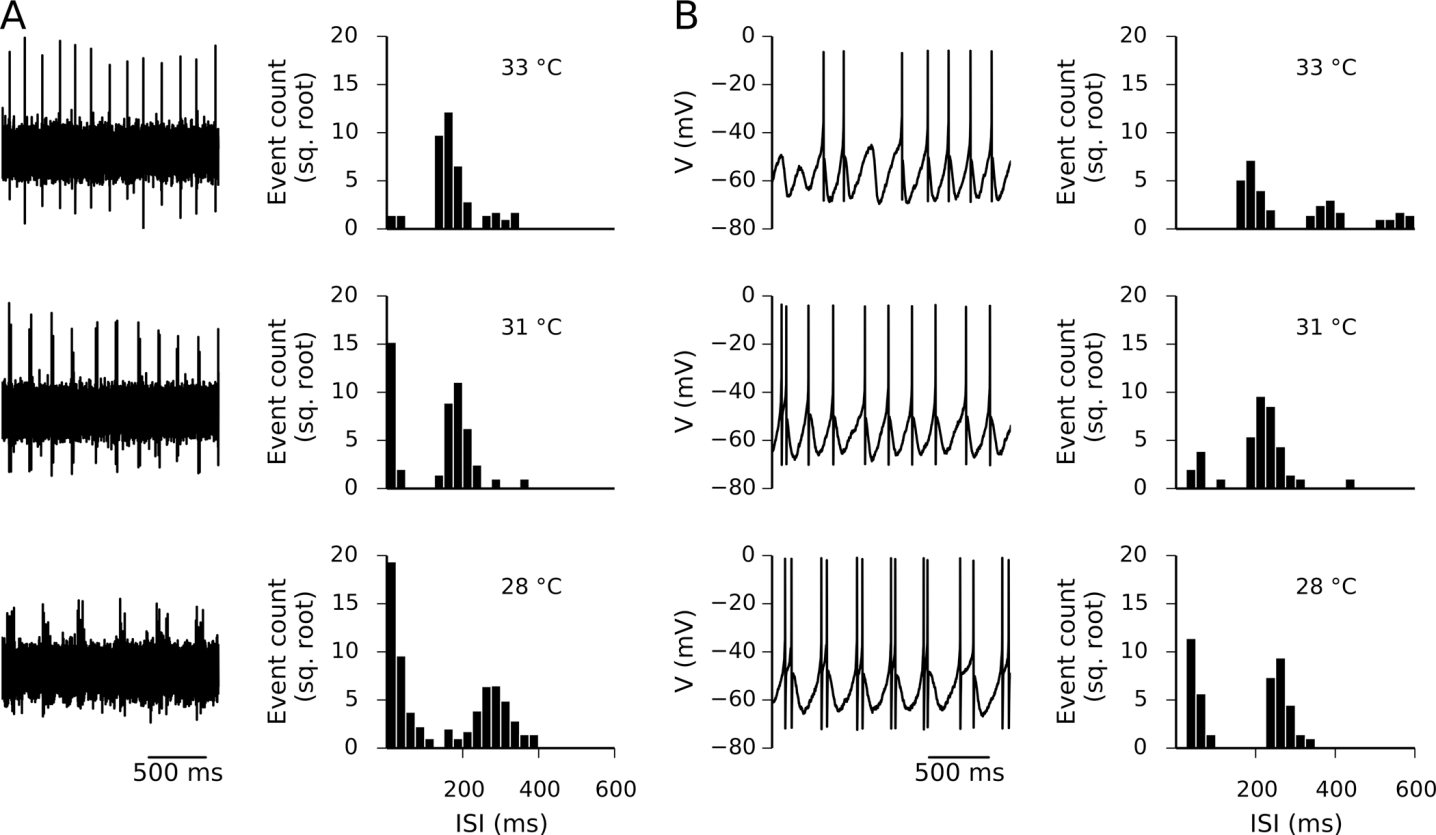
Analysis of the voltage traces and ISI histograms in an experimental recording and the proposed model. A. 2-second extracellular voltage traces (left) and ISI histograms (right) obtained at 3 temperatures from a cold-sensitive nerve ending from mouse cornea. For the histograms, an interval of 60–80 seconds was considered. B. 2-second voltage traces (left) and ISI histograms from the model with parameter set 185.

Cold-sensitive nerve endings not only have a distinctive response to changes in temperature (the dynamic response); they also show different spiking patterns at constant temperatures once the terminal is adapted and the dynamic response vanishes [3,4]. These firing patterns include irregular tonic, regular tonic, and bursting, as well as mixtures of them. While the original models of cold receptors were aimed to reproduce this feature, all bursting features could have been lost by the addition of a new conductance and the manipulation of others parameters. Fig 3A shows extracellular recording from a cold sensitive nerve ending after adaptation to three steady temperatures. As shown in the recordings and the ISI histograms, as the temperature decreases there are more bursting events (seen in the histograms as ISI < 50 ms). At the same time, the period of the fundamental oscillation displaces from a little over 100 ms at 32°C to above 200 ms at 28°C (further statistics of these patterns can be found in [16]). Fig 3B shows that our model also reproduces this characteristic. The ISI histograms closely resemble the experimental ones, as the original model did. This variety of firing patterns is present also at lower temperatures (see below).

**Fig 4:**
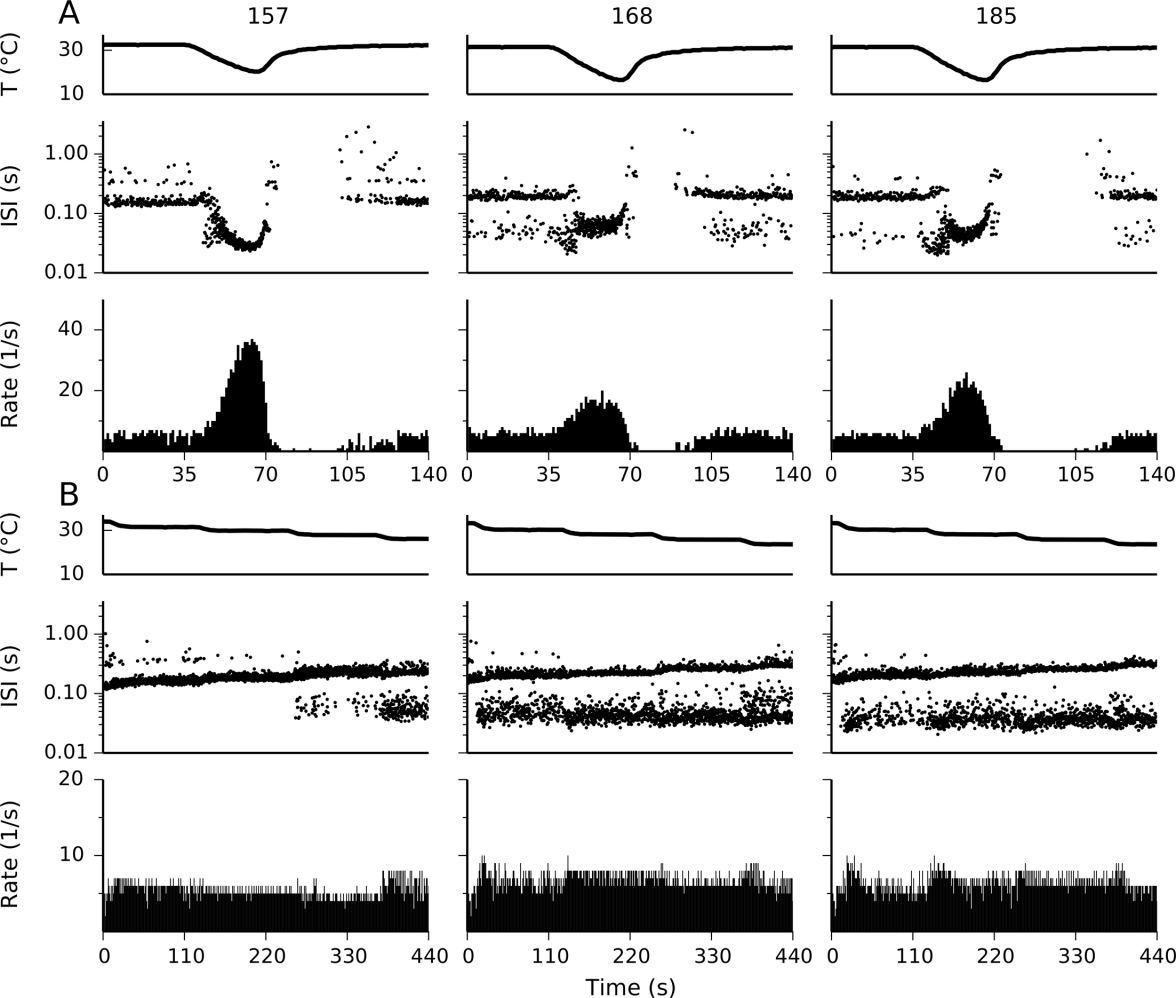
Dynamic and static response of three different set of parameters. Responses to an acute cold pulse (A) and to cooling steps (B) with three different sets of parameters (indicated at the top). Parameters are listed in Table 1. In B, the temperature steps are 31.5°C, 30.0°C, 28.0°C and 26.0°C.

In the search for parameters that allowed the model to reproduce the dynamic response of cold thermoreceptors, we used a rather loose specification of the objectives to be minimized. Thus, it was not surprising that a big number of unrelated parameter sets fulfilled the criteria. Some of them lost the static response properties, but many other retained them. 20 sets of free parameters that reproduce both the dynamic and static response are listed in Table 1. Three examples of different responses are shown in Fig 4. Set 157 produces a stronger dynamic response to the cold pulse shown, while set 168 shows the weakest of the three. They also show different profiles in the static response (Fig 4B), with 157 displaying bursts only at the lowest temperatures and 185 showing strong bursts at all temperatures shown. Looking at the 20 selected parameter sets, we found no obvious correlation between the characteristics of the dynamic and the static responses. As an example, the set with the most prominent bursting (185) has an intermediate dynamic response, while the set with the weakest dynamic response (168) shows an intermediate bursting behavior.

**Fig 5:**
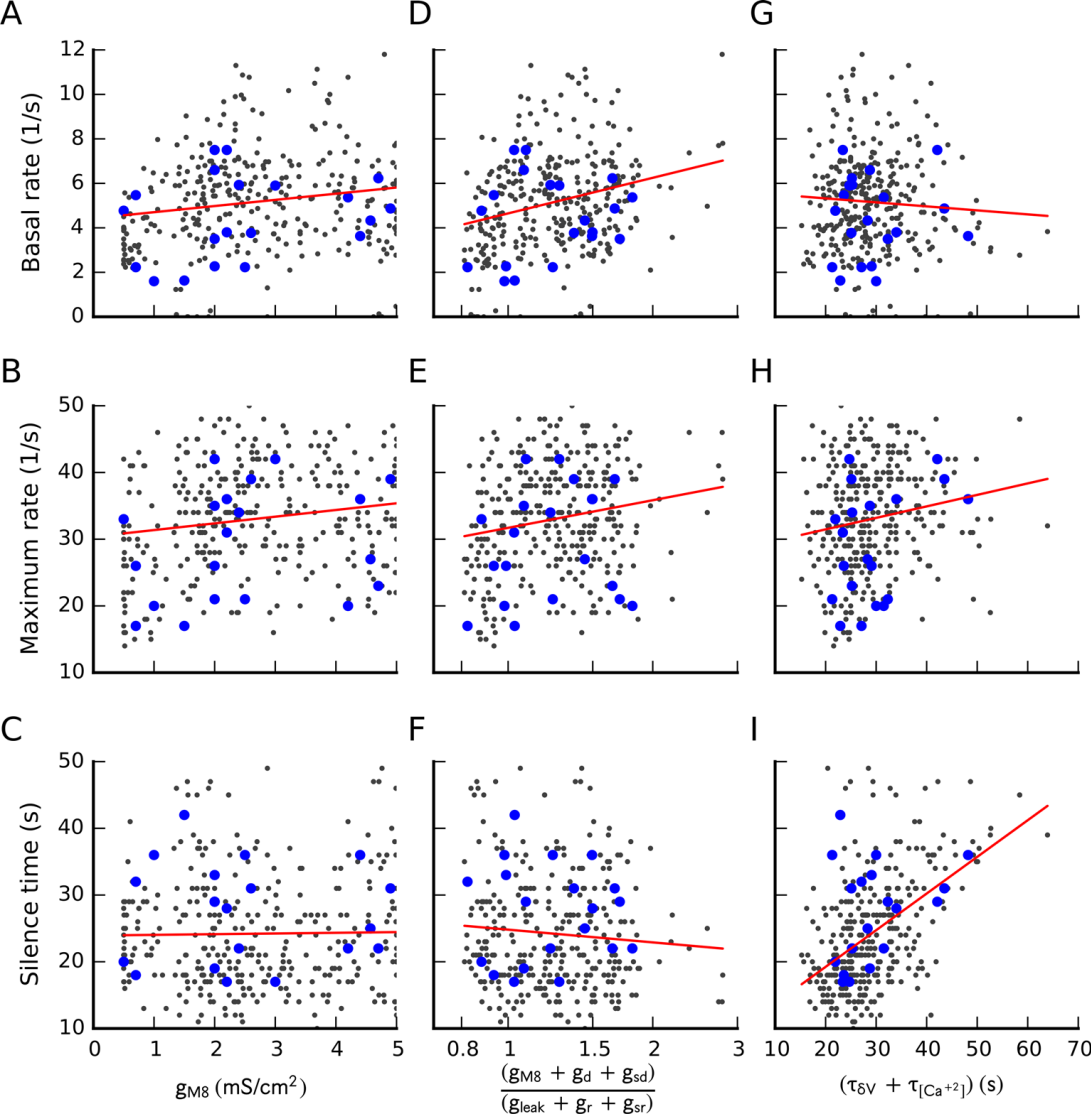
Relationship between some model parameters and the dynamic response. The basal firing rate at 33.5°C (A, D G), the maximum firing rate during the response to cold (B, E, H) and the seconds of silence after the cold pulse (C, F, I) are plotted against the TRPM8 conductance density (A-C), the ratio between depolarizing and repolarizing conductances (D-F) and the sum of the time constants involved in TRPM8 adaptation (G-I). Small black points represent the whole set of 360 parameter sets obtained and the large blue dots correspond to parameter sets listed in Table 1.

We were also unable to find correlations between model parameters and the characteristics of the dynamic response. As shown in Fig 5, neither the basal firing frequency at 33.5°C, the maximum firing frequency during the response nor the length of the silence during the heating phase are correlated with the TRPM8 conductance density or the balance between depolarizing and repolarizing conductances. The only correlation we found was between the length of the silence period and the sum of the time constants governing the activity-dependent desensitization of TRPM8, *τ*_*δV*_ and *τ*_*Ca*_. We did not find any simple correlation between these measures and other parameters (not shown).

**Fig 6:**
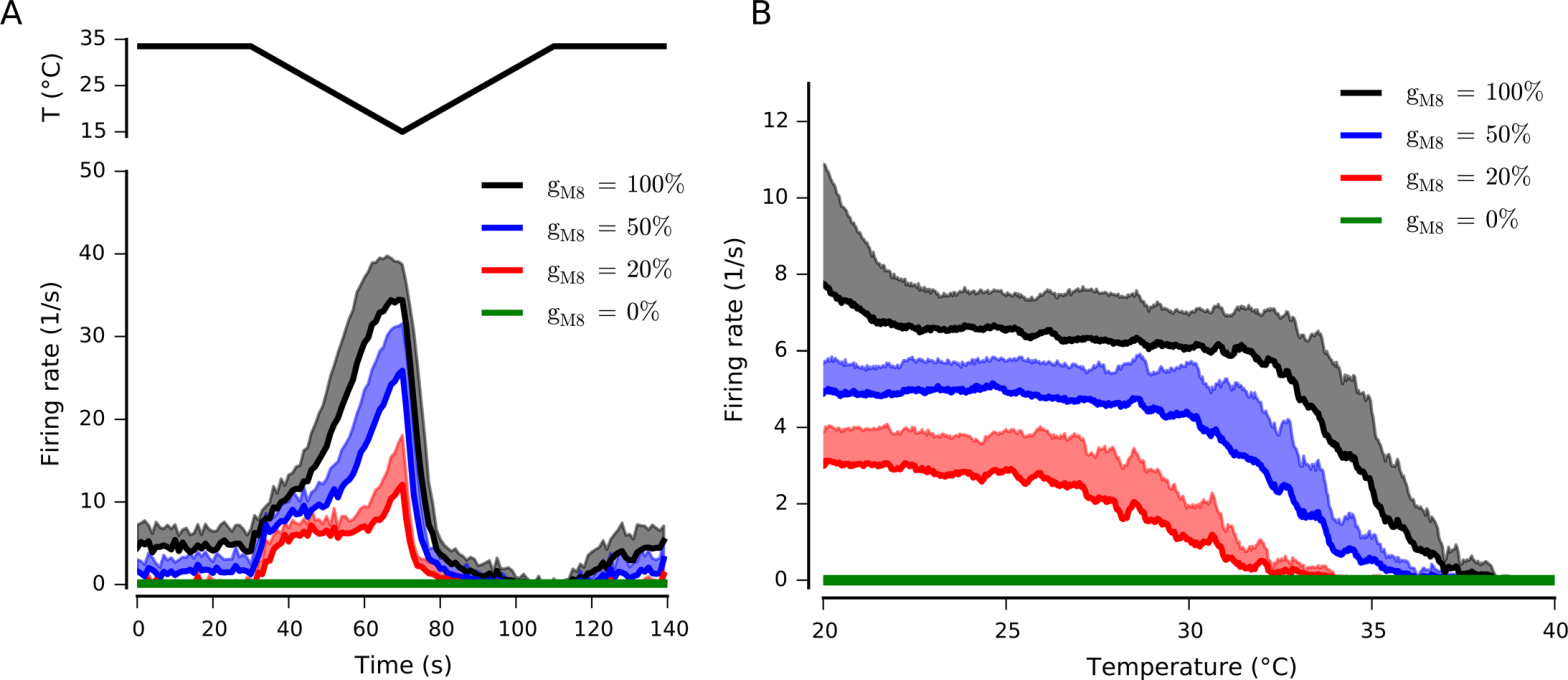
Effect of TRPM8 conductance on firing rate in a cold receptor model. A. Dynamic response of the model with decreasing TRPM8 current density. The trace at the top shows the temperature protocol. The thick lines represent mean firing rate for 20 parameter combinations (Table 1), and the shadowed regions represent standard deviation (only positive value is shown). B. Mean firing rate (thick lines) and standard deviation (shadowed regions) of the static response for the 20 parameter combinations. The static response was obtained simulating a slow temperature ramp (0.033°C/s). Both the dynamic and the static responses show a strong dependence on the TRPM8 conductance density, with no action potentials in the absence of TRPM8.

The activity of cold-sensitive nerve endings is highly dependent on the presence of the TRMP8 channel. Mice lacking the TRPM8 gene are not only devoid of cold-evoked responses in the cornea but they also lack nerve endings with spontaneous activity at basal temperature at the surface of the eye [30]. Therefore, we expect that our mathematical model would also reproduce this feature. Fig 6A shows that the basal firing rate of the model at 33.5°C depends on the maximal density of TRPM8 conductance, *g*_*M*8_. When the maximal conductance is reduced to 50%, so does (approximately) the basal firing rate, and when the conductance is 20% or lower there is no basal (spontaneous) activity. The maximal response to a cold pulse is also diminished when the conductance decreases. Notably, in the absence of TRPM8 there is neither basal activity nor response to the cold stimulus as low as 20°C. Fig 6B shows that the TRPM8 conductance density affects the spontaneous (static) firing rate at all temperature ranges, implying that TRPM8 not only sets the acute response to cold pulses but is also a key player in setting the static response at different temperatures. Note that data presented in Fig 6 corresponds to the average behavior of the 20 parameter sets presented in Table 1.

**Fig 7:**
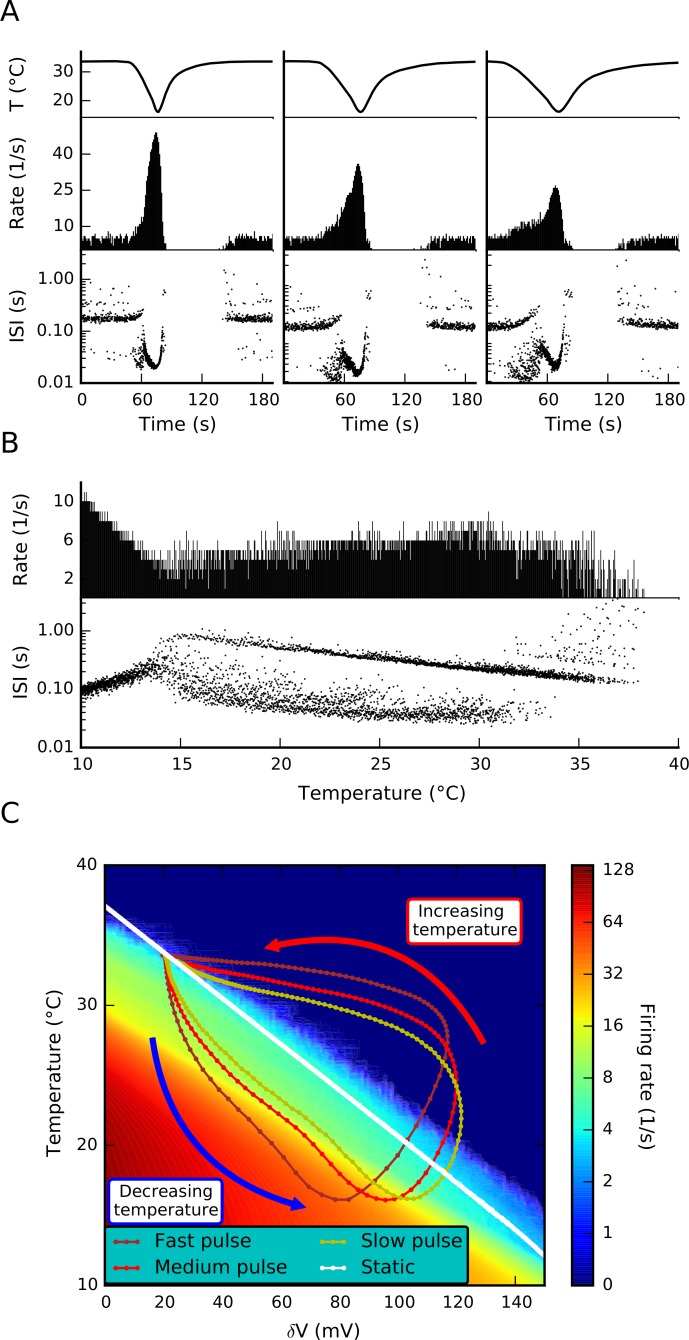
Mechanism of hysteresis in the response to cold. A. Temperature protocol (top), firing rate (middle) and inter spike intervals (bottom) for fast, medium and slow cooling pulses. B. Firing rate and inter-spike intervals during a static response protocol, obtained with a very slow temperature ramp (-0.033°C/s). Also, Ca^2+^ and δV dynamics were accelerated by a factor of 50. C. Firing rate at different degrees of TRPM8 adaptation (δV) and temperatures. Color scale represents firing rate in spikes/sec. The colored lines depict the trajectory of δV and temperature during the protocols shown in A. The white line shows δV versus temperature for the static (adapted) response (B).

Cold-sensitive nerve endings are known to be detectors of temperature changes rather than temperature itself [3]. Thus, their response to an acute temperature drop depends not only on the pulse magnitude but also on the rate of temperature descent. This is illustrated with our model in Fig 7A, where the response is shown for fast (-1.2°C/s), medium (-0.8°C/s) and slow (-0.6°C/s) cold pulses. While the fast pulse evokes a response near 60 impulses/s, the slow pulse barely duplicates the basal rate reaching about 15 impulses/s. To illustrate the static response, Fig 7B shows the behavior of the model during a very slow temperature ramp. To ensure that the model activity is in a steady state with respect to TRPM8 desensitization, Ca^2+^ and δV dynamics were accelerated by a factor of 50. As reported previously for cold thermoreceptors [3], there is a wide temperature range (15–35°C) for which the firing rate is mostly constant and, if any, shows an inverted U dependency.

The hysteresis in the response to a cold pulse is further examined in Fig 7C. We fixed the value of δV, the desensitization variable for TRPM8 current, and measured the average firing rate of the model within the 10–40°C temperature range (shown in color scale). There is a diagonal in the graph, given by the fact that as δV is increased the model starts firing at lower temperatures. In the static response, a state in which the TRPM8 desensitization is at equilibrium for a given temperature, the model is situated almost at the border of the no-firing zone. During the response to a rapid temperature drop, the model is taken away from this equilibrium zone into an area of high firing rate. As desensitization (increase of δV) starts to occur, the model transits to a lower firing rate. The balance between the rate of temperature descent and the kinetics of TRPM8 adaptation sets the maximum firing rate to be achieved. Conversely, while re-heating to the control temperature the model is taken to the no-firing zone and the balance between heating rate and the desensitization kinetics sets the amount of time to be spent in the silence zone.

Thus, by taking in consideration the known desensitization of the TRPM8 current during cold stimulation, our model reproduces the features of the dynamic response to cold observed in cold thermoreceptors.

## Discussion

Detection of changes in the environment is the most important task of sensory systems. In the visual system, for instance, the most relevant information conveyed is about edges, contrast and movement [39], which are nothing but changes within an otherwise constant background. Moreover, a great part of these features are detected as early as in the retina [40]. In olfaction, primary sensory neurons are able to codify small changes in concentration of odor stimuli, in a process that begins with the activation of a large family of receptors expressed in olfactory cilia [41]. Temperature sensation is not a exception to this, as evidenced by the fine detection of temperature changes observed in cold thermoreceptors [2,3]. How changes (sometimes small changes) are detected and reported to the CNS more vividly than constant environment conditions is an important question in the field of sensory systems. Here we propose a mechanism for the detection of temperature changes in cold thermoreceptors that work in the innocuous temperature range. Based on the cold-evoked depolarizing current carried by TRPM8 and its calcium-dependent desensitization, our model shows how the slow time scale of the desensitization is the basis for the detection of changes and the hysteresis of the response.

Intracellular calcium modulates neural excitability at several levels, from the rapid onset of a calcium-activated potassium current that hyperpolarizes the cell [42] to a long-term change in channel expression [43–46]. Common to all these phenomena is the concept of homeostasis, where calcium exerts or is part of a negative feedback mechanism that allows the neuron to self-regulate its excitability. Thanks to this mechanism, in our model the adapted firing frequency maintains a low value (4–8 impulses per second) throughout a wide temperature range, from 15–20°C to 35°C (see Figs 6B and 7B). It is important to note that we did not look for this feature in the parameter fitting procedure; only the basal (33.5°C) firing rate was constrained. The negative feedback that we set up in the equations resulted in the maintenance of a low (static) firing rate over a large temperature range. This allows for a rapid response upon a temperature decrease, no matter what the starting temperature is.

Having an almost constant firing rate from 20°C to 35°C does not mean that the electrical activity is the same. Our model maintains a key feature of both cold thermoreceptors [3] and the original models of static response [6,8], namely, the switching between different firing patterns that range from irregular tonic to bursting (Figs 3 and 4B). This is another feature that was not specified in the constrains for parameter search. Although some of the solutions that we found did not display a robust bursting behavior, they were a minor proportion within the whole population.

Our model accounts for another experimental feature that was not specified in the constrains for parameter search: the dependency of the spontaneous firing activity on the presence of TRPM8, as reported in [30]. Interestingly, heterozygous TRPM8^+/-^ mice display in average a lower frequency of spontaneous activity. This suggests that genetic load is related to channel density and that this in turn determines the acute response of cold sensitive nerve endings as well as their basal activity level. The similarity between Fig 6A in this paper and Fig 2A (left panel) of Parra and coworkers [30], is not limited to *Trpm8* ^*+/+*^ (similar to 100% g_M8_) and *Trpm8* ^-/-^ (similar to 0% g_M8_). In addition, *Trpm8* ^*+/-*^ is comparable to 50% g_M8_, in line with the idea that half the genetic load implies half the channel expression, and that this determines about half of the spontaneous firing rate.

Instead of focusing on a single parameter set that produced a single behavior, we found many satisfactory solutions and present several of them in this work. In doing so, we account for the observed biological variability. In our hands and in others, there is a range of values of basal firing rates, maximum response to cold, characteristics of the bursts in the static response and many other features of the activity recorded from corneal cold thermoreceptors [30,38]. This variability further increases when the observations are extended to other preparations such as lingual, facial or skin nerves [3,5,47]. Our model can be tuned to reproduce several kinds of thermoreceptors that differ either quantitatively or qualitatively. In line with that, we present some results as a population behavior (e.g., Figs 5 and 6) in the same way as experimental results are presented. Also, we used different parameters sets in Figs 1, 2 and 3 selecting those that resembled better the particular experimental result presented. As they were obtained in different experiments, quantitative differences are expected. Importantly, the qualitative features of dynamic and static responses are reproduced by all parameter sets. Unfortunately, we were unable to extract simple correlations between parameters and features. This is probably due to the complex compensations between different parameters that occur in such high-dimensional non-linear models.

In spite of all the experimental features reproduced, our model is far from being exhaustive. In line with the original Huber & Braun’s model for the static response [6], we neglected the inactivation of sodium channels (both fast and slow) and assumed that the slow depolarizing current is a mixture of sodium and calcium channels. Also, we did not use a more complete model, recently published, that includes an hyperpolarization-activated current critical for setting the right oscillation period [16]. We did that in order to test the idea that the original model with the TRPM8 current added and only minimal modifications would be able to reproduce the dynamic response. Most likely, this principle will hold with more complete models, after some parameter adjustments. Further work and refinement of this model will shed light on the complex interactions between the several molecular actors involved in cold thermoreception.

## Supporting Information

S1 Experimental Data Compressed file containing spike trains of CSNTs from mouse cornea subjected to acute cold pulses. Includes figures and summary table.
(ZIP)
