# Supplementary figures and images for: TRPM8-Dependent Dynamic Response in a Mathematical Model of Cold Thermoreceptor
Source: PLoS One. 2015 Oct 1;10(10):e0139314. doi: 10.1371/journal.pone.0139314 (PMC4591370; doi:10.1371/journal.pone.0139314)

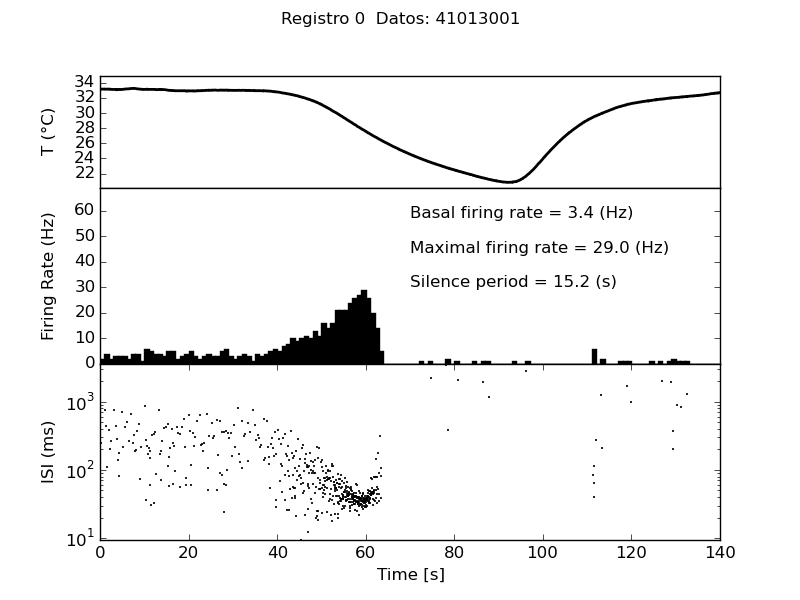

Supplement: S1 Experimental Data — Includes figures and summary table. (ZIP) [file pone.0139314.s001.zip › SupplMat1/pulses_figures/141013001.png]

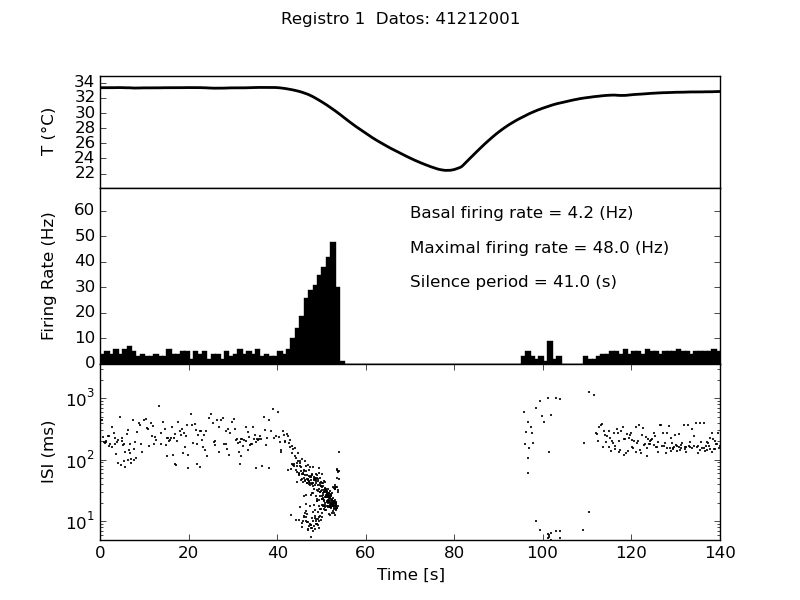

Supplement: S1 Experimental Data — Includes figures and summary table. (ZIP) [file pone.0139314.s001.zip › SupplMat1/pulses_figures/141212001.png]

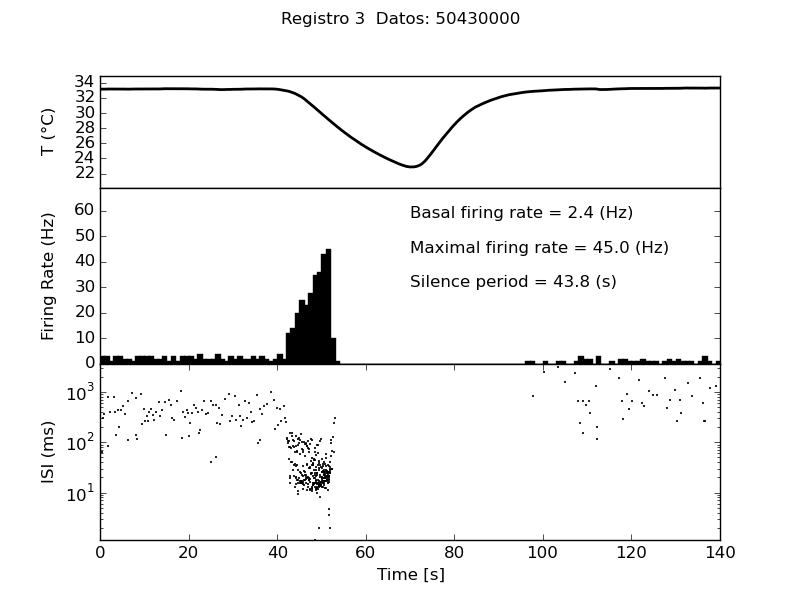

Supplement: S1 Experimental Data — Includes figures and summary table. (ZIP) [file pone.0139314.s001.zip › SupplMat1/pulses_figures/150430000.png]

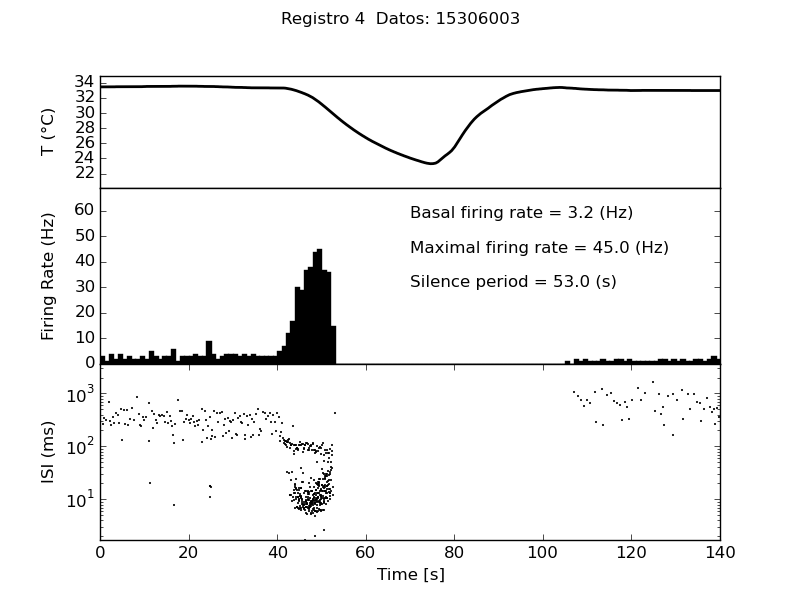

Supplement: S1 Experimental Data — Includes figures and summary table. (ZIP) [file pone.0139314.s001.zip › SupplMat1/pulses_figures/15306003.png]

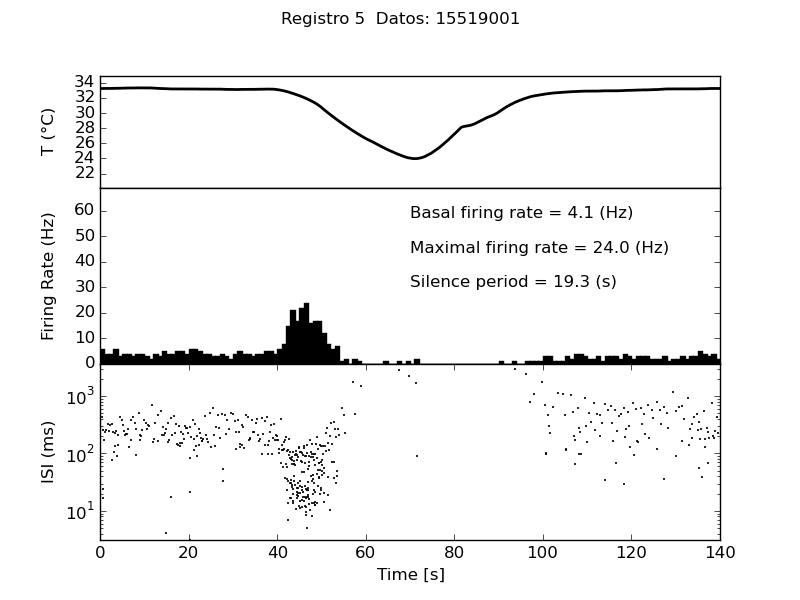

Supplement: S1 Experimental Data — Includes figures and summary table. (ZIP) [file pone.0139314.s001.zip › SupplMat1/pulses_figures/15519001.png]

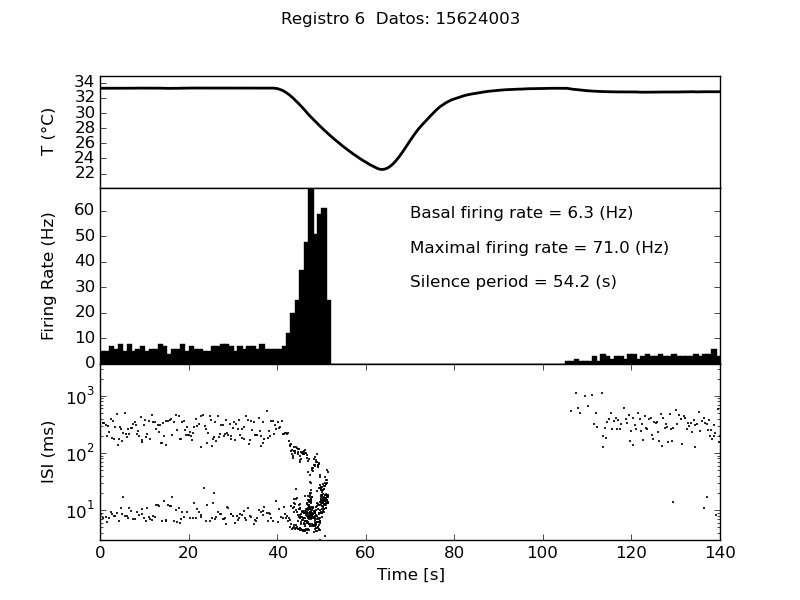

Supplement: S1 Experimental Data — Includes figures and summary table. (ZIP) [file pone.0139314.s001.zip › SupplMat1/pulses_figures/15624003.png]

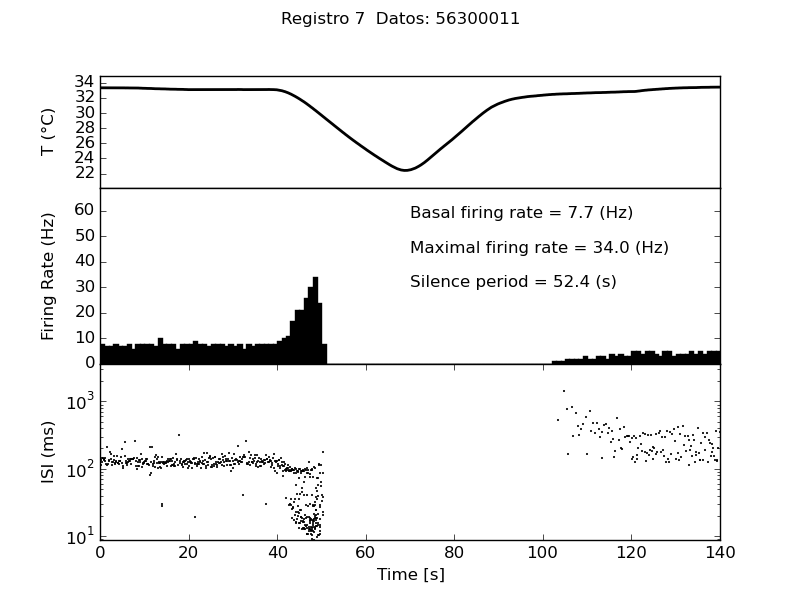

Supplement: S1 Experimental Data — Includes figures and summary table. (ZIP) [file pone.0139314.s001.zip › SupplMat1/pulses_figures/156300011.png]

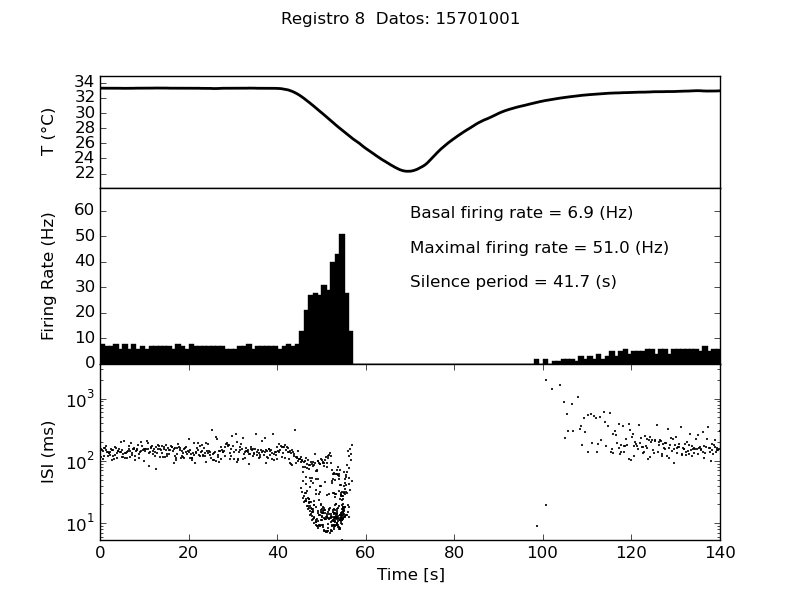

Supplement: S1 Experimental Data — Includes figures and summary table. (ZIP) [file pone.0139314.s001.zip › SupplMat1/pulses_figures/15701001.png]

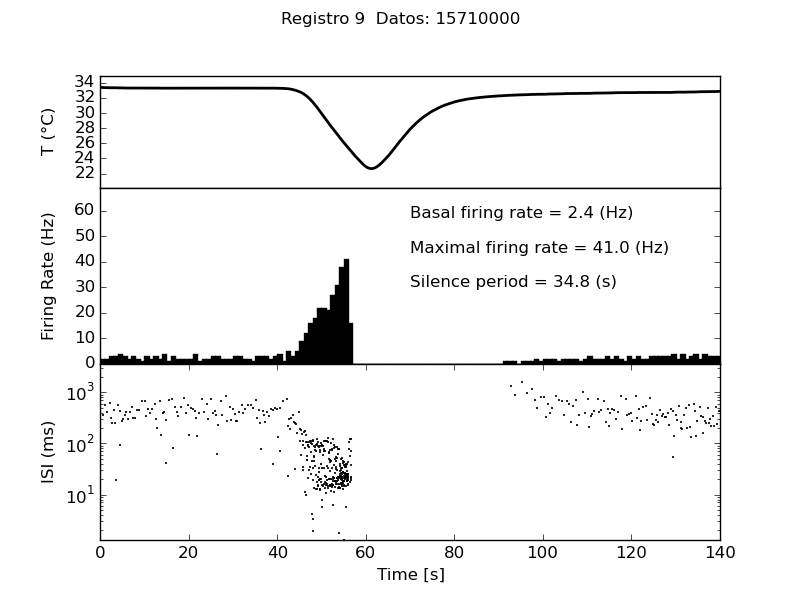

Supplement: S1 Experimental Data — Includes figures and summary table. (ZIP) [file pone.0139314.s001.zip › SupplMat1/pulses_figures/15710000.png]

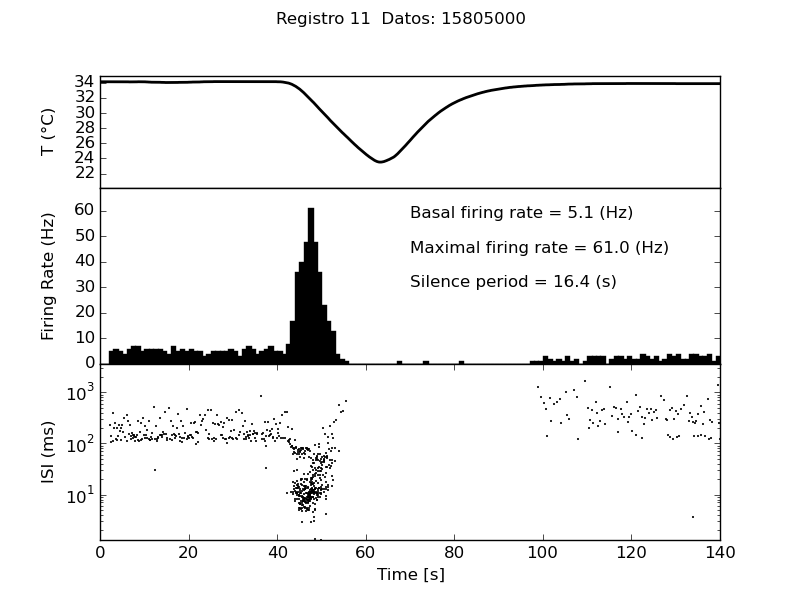

Supplement: S1 Experimental Data — Includes figures and summary table. (ZIP) [file pone.0139314.s001.zip › SupplMat1/pulses_figures/15805000.png]

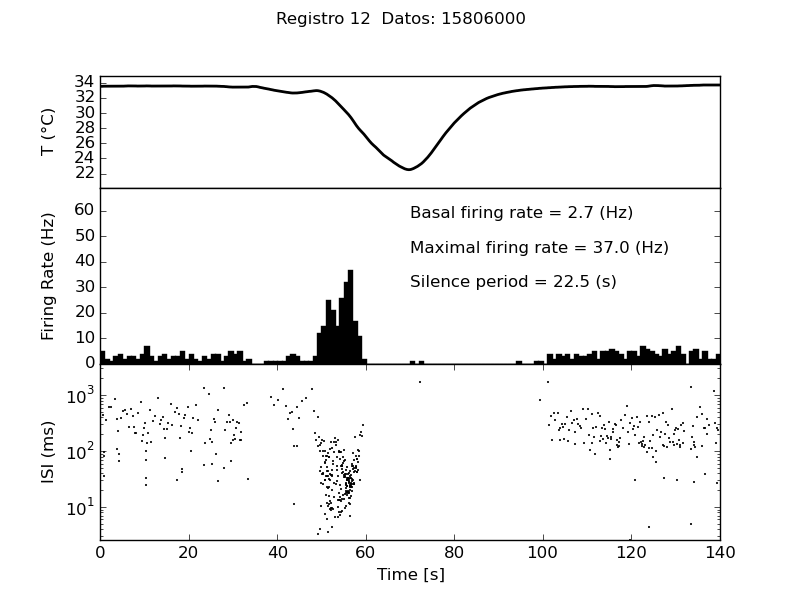

Supplement: S1 Experimental Data — Includes figures and summary table. (ZIP) [file pone.0139314.s001.zip › SupplMat1/pulses_figures/15806000.png]
